# Engaging Patients *via* Online Healthcare Fora: Three Pharmacovigilance Use Cases

**DOI:** 10.3389/fphar.2022.901355

**Published:** 2022-06-03

**Authors:** Greg Powell, Vijay Kara, Jeffery L. Painter, Lorrie Schifano, Erin Merico, Andrew Bate

**Affiliations:** ^1^ GSK, Durham, NC, United States; ^2^ GSK, London, United Kingdom; ^3^ College of Pharmacy, Northeast Ohio Medical University, Rootstown, OH, United States

**Keywords:** adverse event reporting, safety signal detection, follow-up report, social media, patient forums, healthcare fora

## Abstract

Increasingly, patient-generated safety insights are shared online, via general social media platforms or dedicated healthcare fora which give patients the opportunity to discuss their disease and treatment options. We evaluated three areas of potential interest for the use of social media in pharmacovigilance. To evaluate how social media may complement existing safety signal detection capabilities, we identified two use cases (drug/adverse event [AE] pairs) and then evaluated the frequency of AE discussions across a range of social media channels. Changes in frequency over time were noted in social media, then compared to frequency changes in Food and Drug Administration Adverse Event Reporting System (FAERS) data over the same time period using a traditional disproportionality method. Although both data sources showed increasing frequencies of AE discussions over time, the increase in frequency was greater in the FAERS data as compared to social media. To demonstrate the robustness of medical/AE insights of linked posts we manually reviewed 2,817 threads containing 21,313 individual posts from 3,601 unique authors. Posts from the same authors were linked together. We used a quality scoring algorithm to determine the groups of linked posts with the highest quality and manually evaluated the top 16 groups of posts. Most linked posts (12/16; 75%) contained all seven relevant medical insights assessed compared to only one (of 1,672) individual post. To test the capability of actively engage patients via social media to obtain follow-up AE information we identified and sent consents for follow-up to 39 individuals (through a third party). We sent target follow-up questions (identified by pharmacovigilance experts as critical for causality assessment) to those who consented. The number of people consenting to follow-up was low (20%), but receipt of follow-up was high (75%). We observed completeness of responses (37 out of 37 questions answered) and short average time required to receive the follow-up (1.8 days). Our findings indicate a limited use of social media data for safety signal detection. However, our research highlights two areas of potential value to pharmacovigilance: obtaining more complete medical/AE insights via longitudinal post linking and actively obtaining rapid follow-up information on AEs.

## Introduction

The collection of spontaneous case reports to evaluate potential adverse events (AEs) induced by drugs or vaccines is paramount for pharmacovigilance, allowing the timely identification of safety issues and enabling appropriate action including changes in prescribing information.

Traditional methods of analyzing spontaneously reported AEs include individual case review as well as aggregate analysis using disproportionality methods. However, spontaneous reporting, with or without disproportionality methods, has well-recognized limitations such as selective or under-reporting and incomplete information, which can delay or even prevent safety signal detection (Bate and Evans, 2009). The use of observational data from healthcare databases has been a common practice for post-marketing safety studies, but only over the last decade have there been attempts at exploring this vast information for signal detection (Platt et al., 2018). Several data mining methods have already been tested to this aim, allowing suitable signal detection in healthcare databases, although challenges still remain in terms of signal multiplicity and prioritization of the most relevant signals (Arnaud et al., 2017).

Another trend observed over the last decade is that reports are increasingly submitted by patients in addition to healthcare providers (HCPs) (Inch et al., 2012; Inácio et al., 2017). More and more of the patient-generated safety data are shared online, via general social media platforms or dedicated healthcare fora, which allow the patients and HCPs the opportunity to discuss and exchange health and medicine-related information (Freifeld et al., 2014; Nguyen et al., 2017; Li et al., 2020; Golder et al., 2021; Rakhsha et al., 2021). AEs actively reported via social media are readily accessible in large volume and has been shown to enable early warnings for some potential adverse drug reactions see e.g. (Chen et al., 2018).

Social media could therefore be utilized to address several of the existing challenges currently found in patient engagement in safety reporting, and it also has the potential to become routine in pharmacovigilance in the near future (Bate et al., 2018), complementing other routine approaches. Beyond its use in quantitative signal detection, social media has the potential to provide quality longitudinal data that can contribute to the evaluation of drug or vaccine-event pairs, by linking posts from the same individuals. However, there are still challenges to the use of social media data for safety signal detection. The challenges include, but are not limited to: 1) overall poor data quality (lacking information for meaningful evaluation of causality and the use of non-medical language), 2) very high probability of data duplication (Tweets and re-Tweets are prime examples), 3) lack of generalizability to other data sources, 4) non-balanced coverage of all drugs and conditions, and 5) the high volume of data which needs to be curated and analyzed in order to be useful for a safety analysis (Sarker et al., 2015; Bate et al., 2018; van Stekelenborg et al., 2019; Lee et al., 2021).

Regardless of the source and type of data, the quality of initial AE reports received by both competent authorities and the marketing authorization holders (MAHs) is often insufficient to perform a proper causality assessment. Most likely, AE reports fail to capture a complex situation, as they tend to focus on the event itself with limited or no information on other events preceding or following the AE. In addition, other potentially relevant elements (such as environmental exposures, diet, smoking/drinking status or social interactions) are rarely available in traditional data sources. Even for individual safety case reports, which have specific formats imposed by regulatory authorities, the elements missing are frequently the ones that are essential for assessing the report. It is therefore necessary to gather as much additional information as possible, prioritizing reports describing unexpected AEs above those describing expected AEs. Some of the reasons for which follow-up on an initial report is needed is that reporters usually cannot distinguish between essential and non-essential information, they lack time to fully complete the report, or the AE is still ongoing at the time of the report. Pharmacovigilance guidelines worldwide (Code of Federal Regulations, 2011; European Medicines Agency, 2017; Health Canada, 2018; Australian Government et al., 2021) state that the MAH is expected to follow up all reports of serious suspected adverse reactions to its products and to obtain comprehensive information where available, but guidance is broad. This makes follow-up activities difficult, and most times unsuccessful. Most reporters do not respond to follow-up requests, or they take a long time to do so. For instance, in a recent evaluation of follow-up activities for 1,000 AE reports obtained from 58 countries, 87% of the requests were not answered and the average time to receive follow-up information was 47.4 days (median = 23 days; range = 1–432 days) (Kara et al., 2021). Bulcock et al. highlighted that patients have a strong willingness to share health-related social media data about AEs with researchers and regulators (Bulcock et al., 2021).

An increase in the volume and complexity of AE reports has been observed over time, and this together with the processes required for their collection, review, analysis, and dissemination generates an increasing workload and burden of reporting (Stergiopoulos et al., 2019). Pooling various sources may increase the volume of safety data without improving–and quite possibly negatively impacting–the effectiveness of safety signal detection (Jokinen et al., 2019). Therefore, it is essential to identify those niche areas where social media has the most positive impact in pharmacovigilance and ultimately, patient safety. In this three-part study, we assessed the value that patient-generated safety data from online healthcare fora bring to pharmacovigilance with respect to its potential for complementing quantitative signal detection, ability to increase the robustness of AE insights shared online, and opportunities to obtain timely follow-up information that is required for proper assessment.

## Methods

### Objectives

We assessed patient-generated safety data from online healthcare fora in three areas of potential importance to pharmacovigilance activities:

1) changes in the relative frequency of AE discussions in support of traditional signal detection
2) utility of longitudinal AE records obtained by linking posts from the same individual
3) ability to actively engage patients to obtain more information about AEs.

### Datasets, Data Processing, Identification of Use Cases, and Analysis

For each of the three experiments corresponding to the study objectives, different datasets and methodology were used, as described below.

#### Changes in the Relative Frequency of AE Discussions in Support of Traditional Signal Detection

In-scope use cases (drug and AE pairs) were first identified as follows:

− Identification of product: to identify contemporary examples, we considered immune modulators that have been approved in the United States (US) since 2010.
− Identification of event: to determine utility for post-marketing activities, we cataloged all AEs that appeared in the initial US product information (USPI) and AEs that were subsequently added to the USPI for these drugs (Supplementary Table S1).
− Inclusion criteria: to be potentially included in this assessment, each AE had to be medically serious, had not to have appeared in the initial USPI at approval, had to be identified post-approval with at least a 3-years gap between approval and the event being added to the USPI, and had to reflect a medical concept that could be articulated by the patient/caregiver in social media.

From the list, five potential use cases were identified. Subsequent feasibility assessments determined minimal discussions for three of the potential use cases which narrowed the list to the following two use cases: denosumab and multiple vertebral fractures following discontinuation and pembrolizumab and immune-mediated skin adverse reactions (which includes Stevens–Johnson syndrome, toxic epidermal necrolysis, exfoliative dermatitis, and bullous pemphigoid).

**FIGURE 1:**
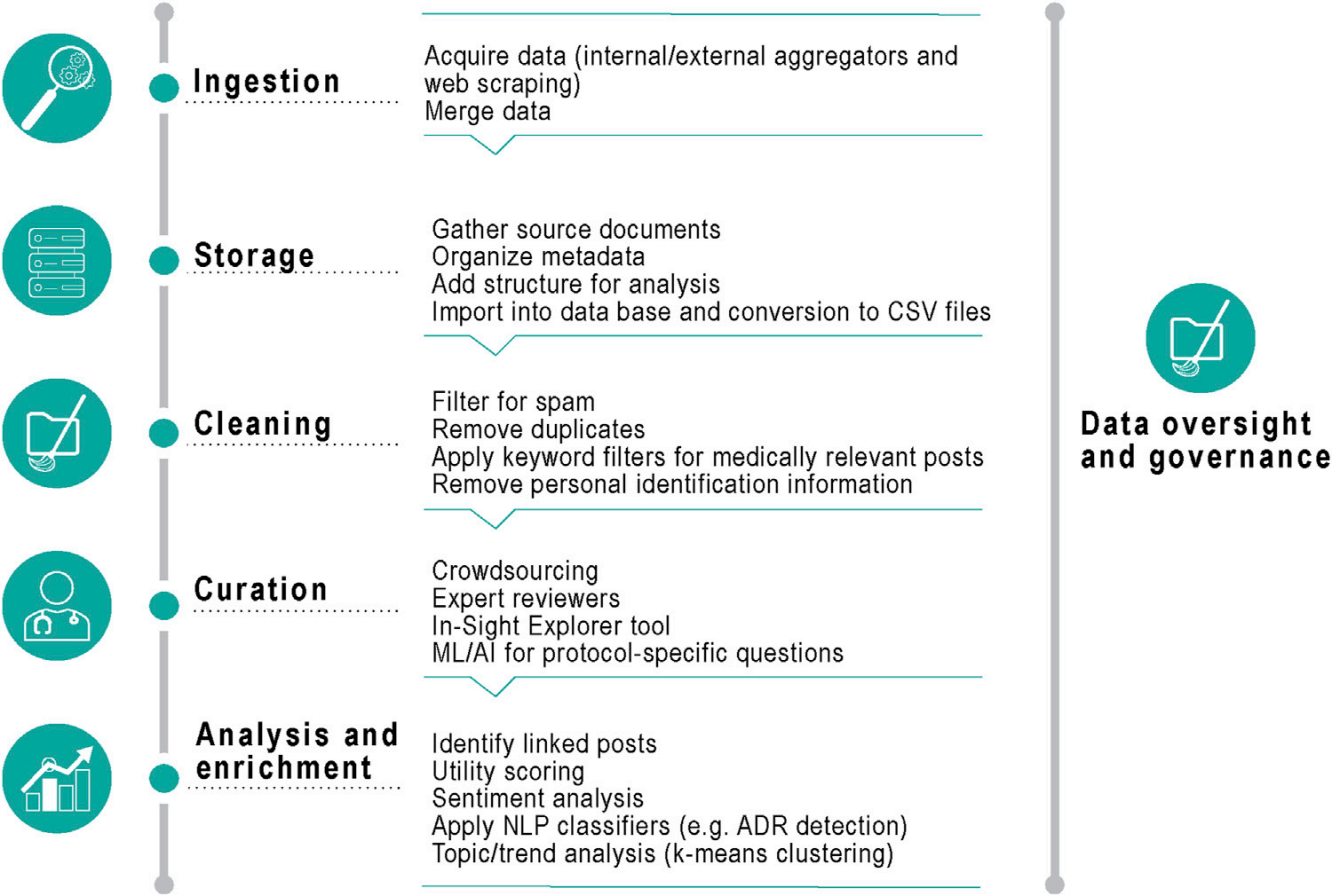
Data processing. CSV, Comma Separated Value; ML, machine learning; AI, artificial intelligence; NLP, natural language processing; ADR, adverse drug reactions.

To go beyond traditional social media, which is primarily focused on social topics, we used data that focused more on healthcare and/or disease discussions. The data sources we used were from four large healthcare communities (Inspire.com and Breastcancer.org, HealthUnlocked.com, and Melanoma.org [through Brandwatch]) and Twitter (through Brandwatch) for contextualization purposes. All posts between June 2010 and April 2021 mentioning denosumab or pembrolizumab were identified and processed according to the steps described in Figure 1, using a combination of Python and Java code. De-identified patient-generated data were retrieved from the third-party social media data aggregator Brandwatch, previously used for the collection of health data from social media (Schachterle et al., 2019; Trigger and Coleman, 2019; Anderson et al., 2022; Gendreau et al., 2022). Brandwatch captures publicly available data from various online platforms or websites and allows one to search across all forums using a set of keywords. Due to the large volume of retrieved data via this method, a subset of all available fora was selected that contained at least 1,000 posts and included mentions of denosumab or pembrolizumab over the observation period of interest (2010–2021).

Duplicate posts (same content, poster, and post date/time) were removed, and a spam removal algorithm was run to look for keywords such as “online store” or press releases (to exclude any non-patient generated data). Medically relevant posts (with at least one mention of the product by generic name, brand name or drug class) were identified by using keyword filters, and all posts were merged into a unified structure suitable for analysis (including meta-data such as a unique post identification number, date, source, content of post and tagged AEs).

Any personally identifiable information was masked from the post content before the curation step, by means of a custom-built dictionary containing over 100,000 common person names and variants. Data review and curation was performed internally. AEs were mapped using the Medical Dictionary for Regulatory Activities (MedDRA) version 24.0 (the latest version available at the time of the analysis) from the Unified Medical Language System (UMLS) (Bodenreider, 2004).

If a post contained any verbatim string that matched an entry in the MedDRA terminology, the UMLS was used to align those terms to the preferred term (PT) level. We developed Python scripts to extract a quarterly summary of the reported AEs from the period indicated and then represented graphically any instance of PTs in the data over time. Individual PTs were then aggregated and collapsed into levels of the MedDRA hierarchy to facilitate data analysis. This allowed rapid identification of MedDRA high level group terms discussed in the posts (Supplementary Figure S1).

Current methods of performing post-marketing signal detection with spontaneous data are focused on identifying disproportionate reporting rates. These methods have been tested with social media with limited results (van Stekelenborg et al., 2019). Rather than using traditional disproportionality methods our approach was more to see if the viral nature of social media discussions might complement traditional methods (Wang et al., 2016).

All posts mentioning the drugs of interest were collected. Counts for the following were computed: 1) total number of posts mentioning the drug of interest and 2) the total number of posts that mention the drug of interest together with any of the AEs of interest (AEOI) and 3) the unique number of posts that mention a drug and a specific AEOI were collected. From these figures, we computed the frequency of each AEOI by dividing the number of events by the total number of posts that mentioned the drug of interest.

To compare the frequency of AEOI discussions in social media to traditional disproportionality methods, safety data from Food and Drug Administration Adverse Event Reporting System (FAERS) for denosumab and pembrolizumab was extracted as PTs of reported AEs (cumulative data as of first quarter 2021) and was reviewed utilizing the empirical Bayes geometric mean (EBGM) methodology (DuMouchel and Pregibon, 2001) for disproportionality analysis within the Commonwealth Vigilance Workbench, Commonwealth Informatics.

#### Utility of Longitudinal AE Records Obtained by Linking Posts From the Same Individual

We obtained publicly available, de-identified social media posts (1 January 2015 to 1 November 2015) from the Scleroderma Foundation Support Community and Arthritis Foundation Support Community fora on Inspire. Each author was given a unique identifier so that posts from the same individual could be linked together longitudinally. Expert reviewers manually curated a random sample of 2,817 threads containing 21,313 individual posts from 3,601 unique authors. To help systematically identify which collection of posts offered the best medical insights we generated a complexity score, comprised of 28 indicators, for each author’s collection of longitudinal posts (Thomas et al., 2017). The group of posts with the 16 highest complexity scores were further evaluated for relevant medical insights through clinical inspection.

For each group of in-scope linked posts the following data elements were manually reviewed and annotated by a healthcare professional: medical history, disease burden, use of non-medical treatments, laboratory results, treatment history, concomitant medications, and mention of AEs. If the post mentioned an AE, other data elements were also annotated: time to onset, outcome, treatment of AE, and statement of causality. The percentages of grouped posts containing each data element were calculated.

#### Ability to Actively Engage Patients to Obtain More Information About AEs

The following steps were used to identify in-scope posts for follow-up:

1. Using Inspire data, we identified all posts that mentioned denosumab (*Xgeva*, *Prolia*) up to 20 April 2021.
2. Posts were de-identified by Inspire before sending them to the study sponsor and unique identifiers were assigned for each person making a post.
3. A custom naïve Bayes AE classification algorithm (trained as described in (Gartland et al., 2021)) was run on the data to identify potential posts of interest.
4. An experienced safety scientist manually reviewed the full list of potential AEs, by descending AE probability score and with a post date of 1 January 2020 or later, to confirm a true AE and temporal relationship to denosumab. False AEs, or those not temporally related to denosumab, were removed from the list. Thirty AEs were selected based on the following, starting with the list of true/temporal AEs:
  i. non-serious/non-severe AEs were removed;
  ii. if no follow-up information was deemed important nor essential for assessment by the reviewer, then the AE was removed;
  iii. of the remaining posts a random sample was chosen (however, a limit of two similar AEs was enforced). If the sample was too small, inclusion criteria were widened;
  iv. only one AE per unique identifier was chosen.
5. Based on the sample of AEs chosen, each post was reviewed independently by two experienced safety scientists and requisite data elements required for proper assessment (defined as containing the minimum information to assess a potential causal association, which may vary by AE) was evaluated and missing data elements documented. Results were compared between reviewers and concordance of relevant missing data elements was achieved. The follow-up attempt sought only the missing data elements.
6. Request for follow-up was sent for the first 15 AEs on the list:
  a. If a person declined to submit follow-up, which was documented, a different AE was chosen.
  b. For those indicating a willingness to submit follow-up information:
    i. after 1 month, if follow-up was not received, additional AEs were chosen for follow-up until 15 completed requests were obtained;
    ii. if follow-up was completed, the individual received an honorarium of $100.00 from Inspire.
7. In the event 15 follow-ups were not received as outlined above, then posts for two other drugs (teriparatide [*Forteo*] or adalimumab [*Humira*]) were collected by Inspire using the same inclusionary dates (1 January 2020 or later) and sent to the study sponsor, and steps above were repeated.
8. After follow-up was received, the reports were reassessed by each safety scientists, and the new assessments were recorded, as well as any comments about the relevancy of the individual data elements provided, whether the follow-up information received now allowed for proper assessment, and the need for further information to properly assess the AE. Concordance across the two assessors was evaluated.

Once the process above was finalized, the study sponsor and Inspire drafted an informed consent form that was to be shared with each participant prior to initiating the engagement. The WIRB Copernicus Group, Inc. (WCG) IRB’s Institutional Review Board Affairs Department reviewed the informed consent and process outlined above under the Common Rule and applicable guidance and determined it exempt under 45 Code of Federal Regulation § 46.104(d) (2), because the research only included interactions involving educational tests, survey procedures, interview procedures, or observations of public behavior and there were adequate provisions to protect the privacy of participants and to maintain the confidentiality of data.

We calculated the percentage of follow-up requests sent and received, time from request to receipt of follow-up, the percentage of cases with at least one (important) piece of information obtained upon follow-up (meaningful follow-up), cases which could be properly assessed after follow-up, and the percentage of cases where all follow-up information requested was obtained.

## Results

### Changes in the Relative Frequency of AE Discussions in Support of Traditional Signal Detection

#### Frequencies of all AEs

In total, 18,934 posts mentioning denosumab and 17,198 posts mentioning pembrolizumab were identified on social media fora. Of these, 13,919 (73.5%) and 9,855 (57.3%), respectively, also mentioned at least one AE which could be mapped to a MedDRA PT code on the same social media posting (Table 1).

**TABLE 1:** Number and percentage of posts mentioning the drugs of interest and at least one adverse event mapped to a MedDRA preferred term, by social media forum and overall.

	Social Media Forum				Total
**Denosumab**	**Breastcancer.org**	**Healthunlocked.com**	**Inspire**	**Twitter**	**—**
N	1159	1113	12588	4074	18934
n	1033	924	10035	1927	13919
%	89.1	83.0	79.7	47.3	73.5
**Pembrolizumab**	**Melanoma.org**	**Healthunlocked.com**	**Inspire**	**Twitter**	**—**
N	1628	971	3257	11342	17198
N	1417	783	2504	5151	9855
%	87.0	80.6	76.9	45.4	57.3

(MedDRA, medical dictionary for regulatory activities; N, number of posts mentioning the drug of interest; n (%), number (percentage) of posts mentioning the drugs of interest and at least one adverse event mapped to MedDRA.

Note: Data from Breastcancer.org, Healthunlocked.com, and Twitter were collected via Brandwatch.

The frequency of the most reported AEs (with ≥2% frequency) grouped by MedDRA PT over the entire period of the study is presented in Table 2 for each drug of interest. A single social media post could contain more than one PT, but if a post mentioned a PT code more than once, it was only counted one time for that post. For denosumab, osteoporosis and fracture were among the PTs most identified: osteoporosis was reported with frequencies between 5.7% and 15.5% across all fora, and fracture with frequencies of 5.5%–6.7% in all fora except Breastcancer.org. Malignant melanoma (4.0%–10.4%), neoplasm malignant (4.8%–18.2%) and neoplasm (5.2%–8.2%) were among the most commonly identified PTs in posts mentioning pembrolizumab (Table 2).

**TABLE 2:** Adverse events (by MedDRA preferred term) reported with frequency ≥2% in posts mentioning the drugs of interest, between January 2010 and December 2021, by social media forum and overall.

—	Social Media Forum			
Denosumab	Breastcancer.org	Healthunlocked.com	Inspire	Twitter
** **N	5828	3433	36178	3034
** **PT (%)	—	—	—	—
—	Surgery (5.7%)	Fracture (6.7%)	Osteoporosis (5.8%)	Osteoporosis (15.5%)
	Osteoporosis (5.7%)	Injection (6.7%)	Fracture (5.5%)	Injection (7.5%)
	Neoplasm malignant (3.8%)	Osteoporosis (6.4%)	Injection (4.7%)	Fracture (6.3%)
	Osteopenia (3.7%)	Pain (3.4%)	Pain (3.9%)	Neoplasm malignant (3.6%)
	Radiotherapy (3.6%)	Blood calcium (3.1%)	Blood calcium (3.4%)	Blood calcium (2.9%)
	Breast conserving surgery (3.4%)	Surgery (2.5%)	Neoplasm malignant (3.0%)	Pain (2.1%)
	Mastectomy (3.3%)	Scan (2.1%)	—	Breast cancer (2.1%)
	Chemotherapy (2.9%)	Prostatic specific antigen (2.1%)	—	—
	Breast cancer (2.5%)	Bone densitometry (2.0%)	—	—
	Injection (2.3%)	—	—	—
	Blood calcium (2.2%)	—	—	—
Pembrolizumab	Melanoma.org	Healthunlocked.com	Inspire	Twitter
N	5479	2593	8221	7317
PT (%)	Malignant melanoma (9.5%)	Prostatic specific antigen (5.9%)	Neoplasm malignant (8.5%)	Neoplasm malignant (18.2%)
	Neoplasm (5.6%)	Neoplasm malignant (5.4%)	Neoplasm (8.2%)	Malignant melanoma (10.4%)
	Neoplasm malignant (4.8%)	Neoplasm (5.2%)	Lung neoplasm malignant (3.6%)	Lung neoplasm malignant (8.9%)
	Infusion (4.4%)	Infusion (4.7%)	Infusion (3.4%)	Neoplasm (5.5%)
	Surgery (4.1%)	Malignant melanoma (4.0%)	Scan (3.2%)	Chemotherapy (4.3%)
	Magnetic resonance imaging (3.7%)	Lung neoplasm malignant (3.7%)	Surgery (2.6%)	Breast cancer (2.4%)
	Scan (3.3%)	Prostate cancer (3.0%)	Malignant melanoma (2.5%)	—
	Positron emission tomogram (3.0%)	Computerized tomogram (2.7%)	Fatigue (2.3%)	—
	Computerized tomogram (2.5%)	Biopsy (2.6%)	Pain (2.0%)	—
	Pain (2.3%)	Fatigue (2.3%)	Chemotherapy (2.0%)	—
	Fatigue (2.0%)	Chemotherapy (2.2%)	Biopsy (2.0%)	—
	—	Positron emission tomogram (2.0%)	—	—

MedDRA, medical dictionary for regulatory activities; N, total number of adverse events (by MedDRA, preferred term) reported in posts mentioning the drug of interest; %, frequency of each adverse event (by MedDRA, preferred term).

Note: Data from Breastcancer.org, Healthunlocked.com, and Twitter were collected via Brandwatch.

#### Frequencies of AEOIs

The following MedDRA PTs were used as AEOI cervical vertebral fracture, lumbar vertebral fracture, thoracic vertebral fracture, and spinal fracture for denosumab and toxic epidermal necrolysis, Stevens-Johnson syndrome, erythema multiforme, dermatitis exfoliative, and pemphigoid for pembrolizumab.

**FIGURE 2:**
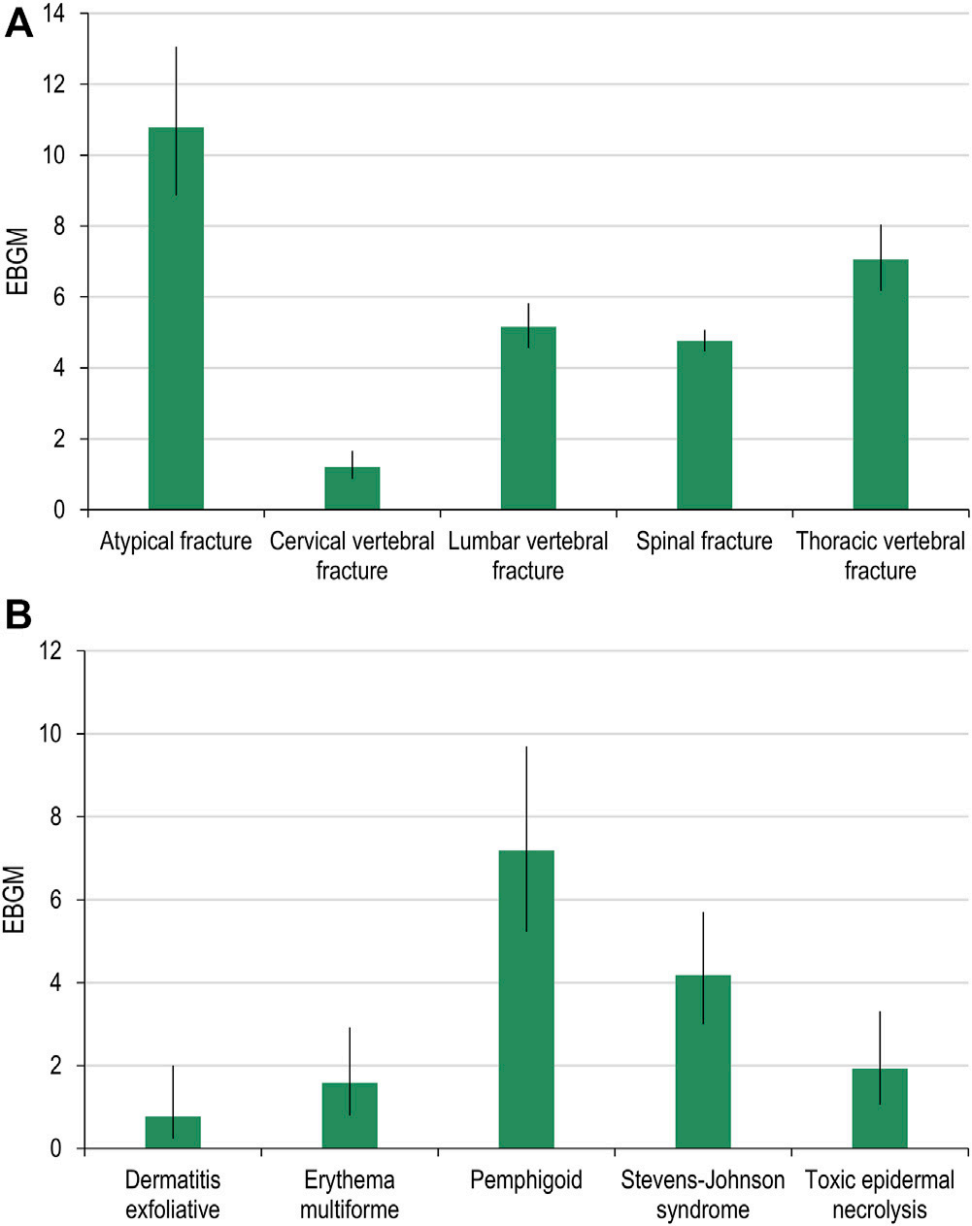
Adverse events of specific interest for denosumab **(A)** and pembrolizumab **(B)** detected in FAERS. FAERS, Food and Drug Administration Adverse Event Reporting System; EBGM, empirical Bayes geometric mean.

For both denosumab and pembrolizumab, AEOIs were identified with varying frequency across social media fora. When considering all social media posts, the frequency of AEOIs ranged from 0.004% to 0.642% for denosumab and from 0.004% to 0.008% for pembrolizumab (Tables 3, 4) at the end of the 10-year period analyzed. The overall number of AEOIs (Tables 3, 4) and EBGM (Figure 2) identified in FAERS are presented for comparison.

**TABLE 3:** Adverse events of interest (by MedDRA preferred term) identified in posts mentioning denosumab.

	Breastcancer.org (N = 5248)	Healthunlocked.com (N = 3433)	Inspire (N = 36178)	Twitter (N = 3034)	Total (N = 48473)	FAERS
	n (%)					n
Lumbar vertebral fracture	—	—	—	—	—	182
Spinal fracture	2 (0.034%)	44 (1.282%)	242 (0.669%)	23 (0.758%)	311 (0.642%)	672
Thoracic vertebral fracture	—	1 (0.029%)	1 (0.003%)	—	2 (0.004%)	154

MedDRA, medical dictionary for regulatory activities; N, total number of adverse events (by MedDRA, preferred terms); n (%), number (relative frequency) of adverse events of interest; FAERS, food and drug administration adverse event reporting system.

Note: Data from Breastcancer.org, Healthunlocked.com, and Twitter were collected via Brandwatch.

**TABLE 4:** Adverse events of interest (by MedDRA preferred term) identified in posts mentioning pembrolizumab.

	Melanoma.org (N = 5479)	Healthunlocked.com (N = 2593)	Inspire (N = 8221)	Twitter (N = 7317)	Total (N = 23610)	FAERS
	n (%)					n
Dermatitis exfoliative	—	—	—	—	—	2
Erythema multiforme	—	—	—	—	—	6
Pemphigoid	—	—	—	2 (0.027%)	2 (0.008%)	28
Stevens-Johnson syndrome	—	—	1 (0.012%)	—	1 (0.004%)	26

MedDRA, medical dictionary for regulatory activities; N, total number of adverse events (by MedDRA, preferred term); n (%), number (relative frequency) of adverse events of interest; FAERS, food and drug administration adverse event reporting system.

Note: Data from Breastcancer.org, Healthunlocked.com, and Twitter were collected via Brandwatch.

Results stratified by quarter are presented in the Supplementary Appendixes S1, S2.

#### Changes in the Frequencies of AEOIs

**FIGURE 3:**
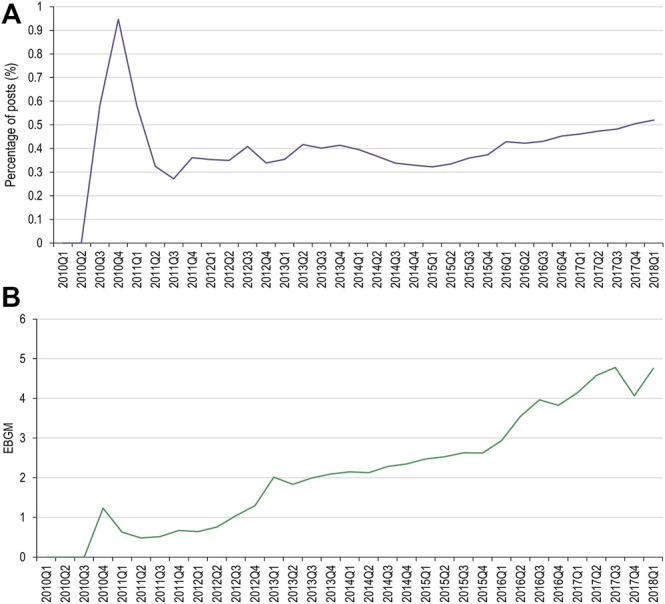
Percentage of social media posts that mention denosumab and spinal fracture as an adverse event **(A)** and EBGM scores for denosumab and spinal fractures from FAERS data **(B)**. FAERS, Food and Drug Administration Adverse Event Reporting System; Q, quarter; EBGM, empirical Bayes geometric mean.

**FIGURE 4:**
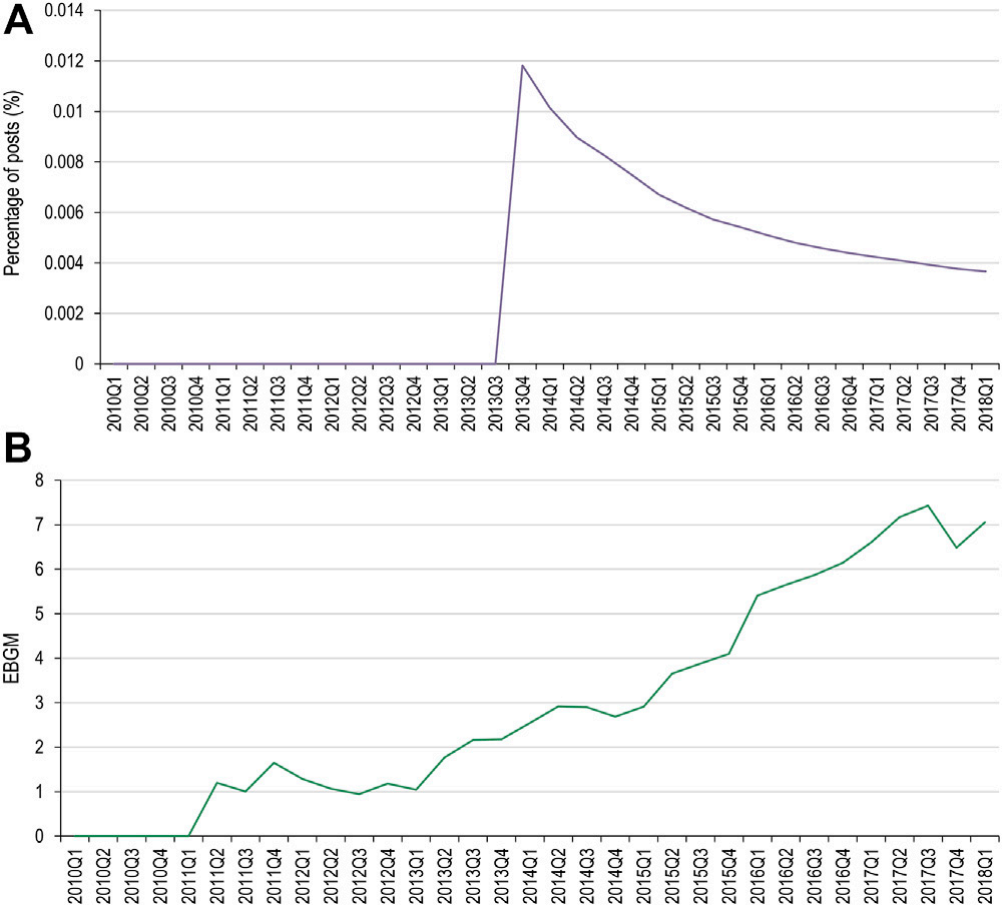
Percentage of social media posts that mention denosumab and thoracic vertebral fracture as an adverse event **(A)** and EBGM scores for denosumab and thoracic vertebral fracture from FAERS data **(B)** FAERS, Food and Drug Administration Adverse Event Reporting System; Q, quarter; EBGM, empirical Bayes geometric mean.

Of the MedDRA PTs used for the denosumab AEOI, cervical vertebral fracture and lumbar vertebral fracture did not have data available in both data sets for comparison. Results for the spinal fracture and thoracic vertebral fracture PTs are presented in Figures 3, 4, respectively. Only spinal fracture had sufficient observations (cumulative count ≥50) in both data sources to reasonably identify frequency changes from product launch until the USPI was updated. The frequency of the AEOI varied from 0.32% to 0.95% in social media posts in 2010 (the first year from approval). The frequencies increased from 0.27% in the third quarter of 2011 to 0.52% in first quarter of 2018 (when the event was added to the USPI) (Figure 3A). A trend of increasing frequencies was seen in the FAERS data, with the EBGM ranging from 0.519 in the third quarter of 2011 to 4.756 in the first quarter of 2018 (Figure 3B).

**FIGURE 5:**
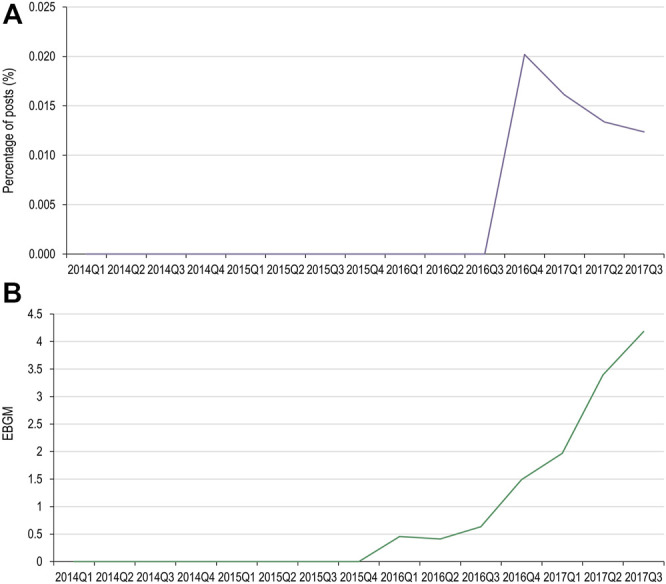
Percentage of social media posts that mention pembrolizumab and Stevens-Johnson syndrome as an adverse event **(A)** and EBGM scores for pembrolizumab and Stevens-Johnson syndrome from FAERS data **(B)** FAERS, Food and Drug Administration Adverse Event Reporting System; Q, quarter; EBGM, empirical Bayes geometric mean.

Of the MedDRA PTs used for the pembrolizumab AEOI, toxic epidermal necrolysis, erythema multiforme, and dermatitis exfoliative did not have data available in both data sets for comparison. Pemphigoid had two occurrences in social media, however both occurred after the USPI had been updated. Stevens-Johnson syndrome had one occurrence (Figure 5).

### Utility of Longitudinal AE Records Obtained by Linking Posts From the Same Individual

A total of 1,672 posts were evaluated, which averaged to 104 posts per author (median 34, range 11–534). Relevant medical insights mentioned by the authors in the 16 posts with the highest complexity scores included medical history (16/16, 100%), disease burden (15/16, 94%), use of non-medical treatments (15/16, 94%), laboratory results (14/16, 88%), treatment history (13/16, 81%), AEs (13/16, 81%), and concomitant medications (12/16, 75%).

Among the posts that contained AE information, additional details were provided such as: outcome of the event (13/16, 81%), time to onset (11/16, 69%), treatment of the event (10/16, 63%), mention of causality (10/16, 63%), dose of the medications (9/16, 56%), and mention of rechallenge/de-challenge (3/16, 19%).

**FIGURE 6:**
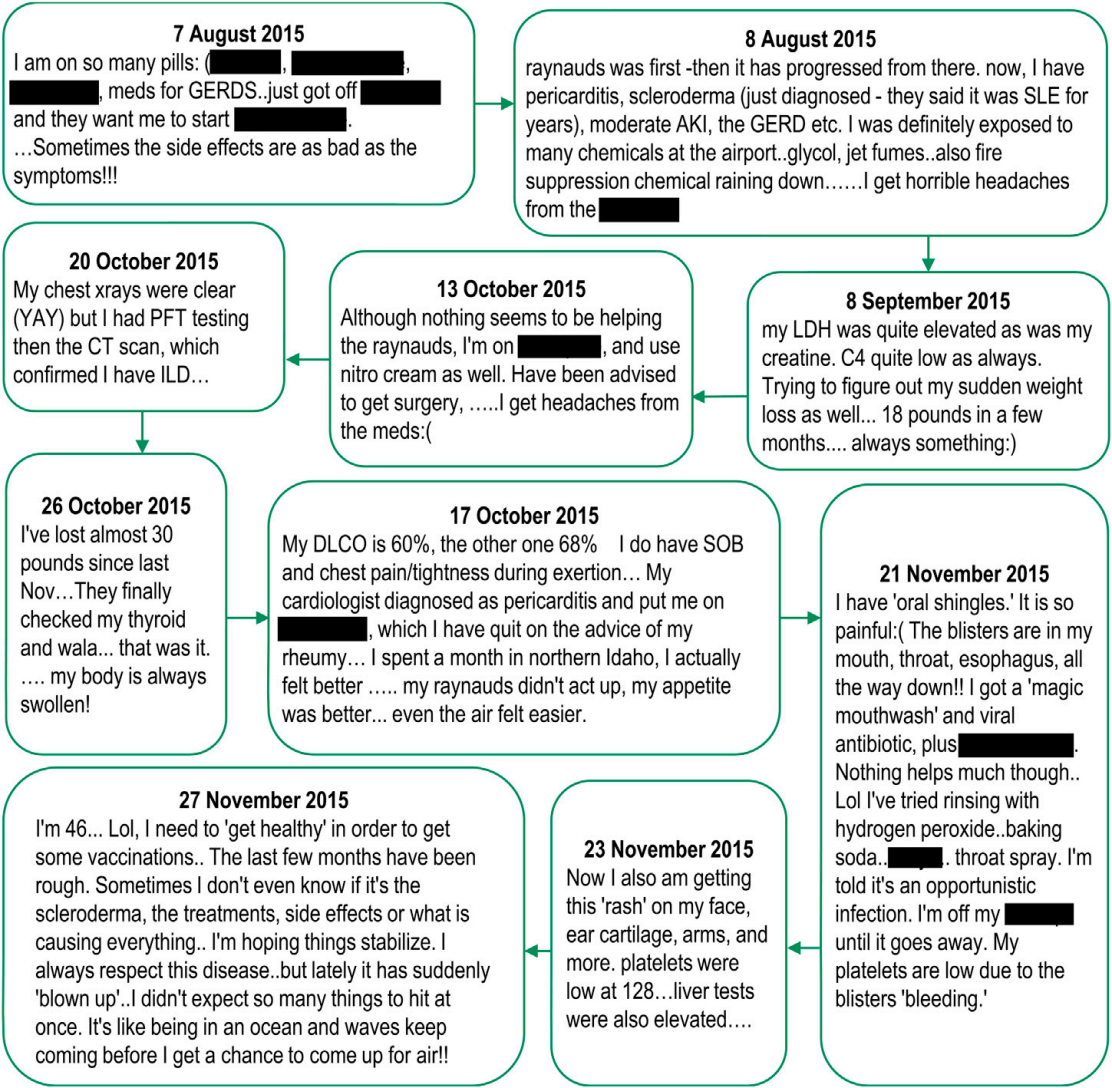
Relevant medical and adverse event insights from spontaneously reported adverse events in social media, posted by a single individual. Note: Posts are listed verbatim but mentions of drug names have been redacted.

Notably, the above insights were seen cumulatively across the various postings of an individual rather than within a single posting (only one post out of the 1,672 contained all seven medical insights above). See Figure 6 for an exemplar of how relevant medical and AE insights can be spread over multiple posts.

### Ability to Actively Engage Patients to Obtain More Information About AEs

Among a subset of Inspire users, 39 requests were sent to follow-up on posts mentioning an AE in relation to one of the drugs of interest. Eight (8/39, 21%) of these requests were answered with a consent for further follow-up, for which six (6/8, 75%) patients provided the information requested. The median time for receipt of follow-up from these patients was 2.5 days (range 0–27 days) from the date of the initial contact seeking consent and 0.5 days (range 0–4 days) from the date the follow-up questions were sent.

Depending on the initial information reported, the request for follow-up contained between four and 11 questions. Most of the questions focused on obtaining further information regarding the diagnosis, duration, and risk factors for the AE, history of similar events prior to treatment, time to onset, concomitant medication and relevant medical history. All questions asked during the follow-up were answered by the initial posters (37/37, 100%). After reviewing the follow-up, the AE was assessable for 4/6 (67%) and non-assessable for 2/6 (33%) of the reports. All patients (6/6, 100%) were willing to share their HCP’s contact information for additional follow-up.

**FIGURE 7:**
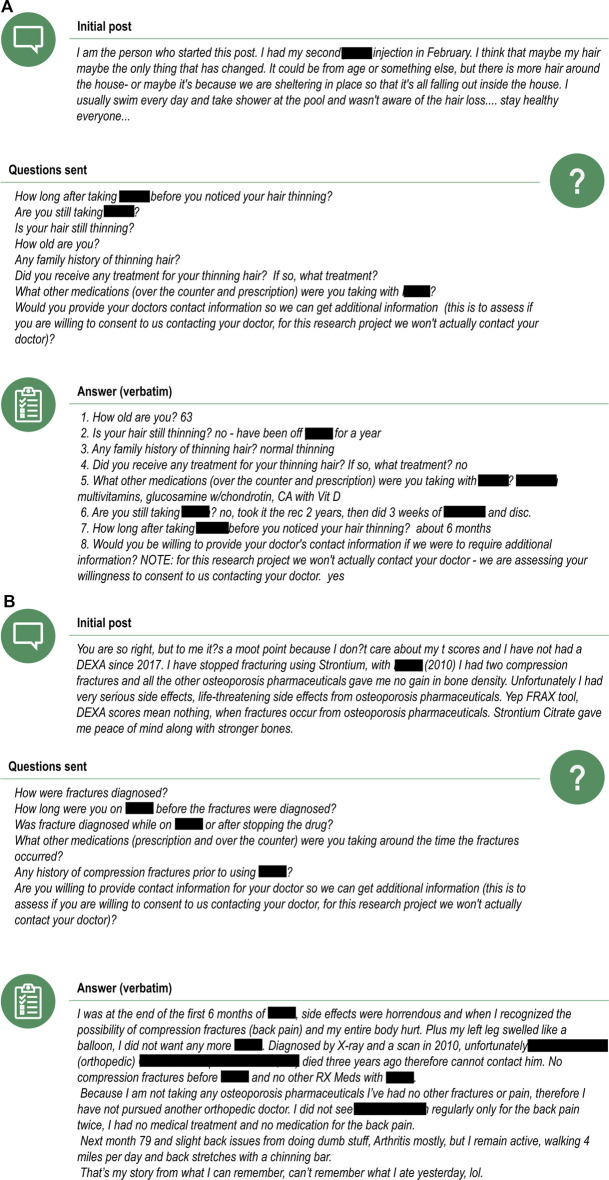
Examples of an assessable **(A)** and non-assessable **(B)** case after follow-up information was requested and provided by the patient. Note: The drug of interest was denosumab. Posts are listed verbatim but mentions to trade names of drugs or sensitive personal information have been redacted.

Examples of initial posts, and information provided after follow-up was requested, are shown in Figure 7, for an assessable (panel A) and a non-assessable (panel B) report after follow-up was obtained. All other reports are presented in Supplementary Figure S2.

## Discussion

The advent of the internet and online communication has broadened considerably the range of data sources available for pharmacovigilance activities, from real-world data from healthcare databases or electronic health records to data generated by monitoring health apps or devices (Bate and Stegmann, 2021). Social media has emerged as a potentially complementary source of safety data in pharmacovigilance, by supplementing data from individual safety case reports or other sources (Sarker et al., 2015), but to date, its full potential remains unknown. We describe three distinct analyses, designed to investigate the complementary role of social media data in pharmacovigilance through trends over time, generating longitudinal safety insights direct from patients, and the possibility of obtaining higher quality follow-up data by actively engaging individuals who mention AEs online.

When evaluating changes in frequency, only one AEOI (denosumab and spinal fracture) had enough data to compare the frequency trends in both social media and FAERS. The range of frequencies of the AEOI in social media posts were highly variable in the first year from approval, but a more consistent trend of increase was observed over the next 7 years, up to the year when the event was added to the USPI. While a similar trend of increasing frequencies was seen in the FAERS data, the magnitude of change over time was substantially greater for the EBGM compared to the social media frequency: 816% increase in EBGM versus 93% increase in social media frequency over the same time period. Based on our very limited data set, our findings do not suggest that social media can highlight emerging issues earlier than spontaneous reports. Our results are therefore consistent with a recent publication by The Innovative Medicines Initiative WEB-RADR (Recognizing Adverse Drug Reactions) consortium, a partnership including members from European regulatory agencies, MAHs, academia and patient groups, who concluded that social media was not recommended for broad statistical signal detection (van Stekelenborg et al., 2019).

When evaluating the utility of longitudinal AE records, we found that the average number of posts per author was high (104 posts, with a minimum of 11 and a maximum of 534 posts per author). Moreover, only one of the 1,672 reviewed posts contained all relevant medical insights, highlighting that information related to the author is disparate across multiple threads and discussion. However, by longitudinally-linking posts, we were able to obtain additional relevant medical insights not available in the primary post. This is the first assessment of the utility of longitudinal AE records for relevant medical/AE insights, and suggests that longitudinal analysis of social media data, when such permissions have been granted, provides insights which cannot be obtained from cross-sectional analysis and is likely to provide complementary insights to those achieved through cross-sectional spontaneous reporting. Makady et al. have similarly noted the value of longitudinal posts in web-based forums in their study assessing quality-of-life information in support of health technology assessments (Makady et al., 2018).

When evaluating the ability to actively engage patients using social media to provide follow-up information for AEs, we found the overall response rates for follow-up requests are quite low, similar to those previously described in the literature for spontaneous reporting [20% (8/39) versus 18% (184/1000)]. However the receipt of follow-up once consent was received was high, also similar to those previously described in the literature for spontaneous reporting [75% (6/8) versus 68% (126/184)] (Kara et al., 2021). The two areas that performed well with respect to actively seeking follow-up via social media were the completeness of response [100% (37/37) of all questions were answered] and the average time required to get the follow-up (1.8 days as compared to 47 days for spontaneous reports (Kara et al., 2021). While the sample size for this part of the study was low, thus limiting the generalizability of the results, these findings indicate the potential of social media in obtaining rapid follow-up information on AEs, noting however that it is important to ensure this is done with the appropriate oversight in place to ensure patient privacy (Li, 2015; Correia et al., 2020). Additionally, participants in our study received an honorarium of $100 from Inspire for completion of the follow-up, and the impact differing amounts of renumerations on the results are unknown.

Our study has several limitations. First, most of our data stem from a few, select disease-specific fora with all posts being made in the English language that may not be generalizable to other data sources and/or other languages. Additionally, using anonymized data did not allow us to correct for duplicate posts. The role of automated techniques for the detection of AEs from social media and its subsequent extraction may lower precision and introduce bias. Second, Brandwatch and other social media channels used in this study might not be able to capture all social media mentions due to privacy settings across platforms. We only used a subset of posts for all analyses and patients consenting to participating in studies may not generalize to wider users of social media. Thus, our data may not be fully representative of all online posts on the drug and the AEOIs. Part of our analysis only focused on two drugs of interest (denosumab and pembrolizumab), belonging to the same drug class, which may limit the generalizability of the results to other drug-AE pairs. Moreover, safety data stemming from social media should be interpreted with caution due to potential veracity issues and the inability of fully understanding the context of a given post. Finally, most of our analyses were performed on small sample sizes, and additional work is needed to see if larger data sets will produce similar results. Our research was meant to highlight some niche areas where social media may be of value, but there are likely other such areas that are still to be explored. A substantial amount of research is still needed to adequately understand the strengths and weaknesses of how social media can support pharmacovigilance in these niche areas.

In conclusion, the use of social media in pharmacovigilance remains to be fully defined. While much research has focused on social media as an alternative and similar data source to spontaneous reports, our research suggests that the potential value of social media may be very distinct and different from traditional spontaneous reports. The obvious key challenge remains determining how social media may complement spontaneous reports in these areas. Although we noted an increase in the frequency in people discussing an AEOI long before it was added to the USPI, we saw a much more pronounced trend in EBGM scores over the same time period. It remains unclear how the increase in frequencies of AE discussions in social media may complement quantitative signal detection. The other two niche areas discussed in our paper may offer more potential value to pharmacovigilance. Linking posts longitudinally offers the ability to potentially generate more robust medical/AE insight and actively engaging patients online may enable rapid, more complete follow-up information for AEs. This is not dissimilarly to other real-world data sources in pharmacovigilance (e.g., medical records, insurance claims databases), although we are aware of the differences stemming from the distinct nature of the sources. The importance of a clear, well-articulated clinical rationale behind the suspicion of an AE is indisputable, and for this reason patient reporting on social media should not be anticipated to replace the role of traditional spontaneous reporting. Nevertheless, as the use of social media has become ubiquitous in younger generations, its value in terms of volume and quantity of data will only increase. Therefore, pharmacovigilance as a field needs to consider the potential value that social media can provide as a complementary source to spontaneous reports.

## Data Availability

The original contributions presented in the study are included in the article/Supplementary Material, further inquiries can be directed to the corresponding author.

## References

[B1] Anderson J. T. Bouchacourt L. M. Sussman K. L. Bright L. F. Wilcox G. B. (2022). Telehealth Adoption during the COVID-19 Pandemic: A Social Media Textual and Network Analysis. Digit. Health 8, 20552076221090041. 10.1177/20552076221090041 35392254 PMC8979849

[B2] Arnaud M. Bégaud B. Thurin N. Moore N. Pariente A. Salvo F. (2017). Methods for Safety Signal Detection in Healthcare Databases: a Literature Review. Expert Opin. Drug Saf. 16 (6), 721–732. 10.1080/14740338.2017.1325463 28490262

[B3] Australian Government, Department of Health, and Administration, T.G (2021). Pharmacovigilance Responsibilities of Medicine Sponsors: Australian Recommendations and Requirements. Available: http://www.tga.gov.au/sites/default/files/190214_pharmacovigilance-responsibilities-medicine-sponsors.pdf (Accessed Mar 16, 2022).

[B4] Bate A. Evans S. J. (2009). Quantitative Signal Detection Using Spontaneous ADR Reporting. Pharmacoepidemiol Drug Saf. 18 (6), 427–436. 10.1002/pds.1742 19358225

[B5] Bate A. Reynolds R. F. Caubel P. (2018). The Hope, Hype and Reality of Big Data for Pharmacovigilance. Ther. Adv. Drug Saf. 9 (1), 5–11. 10.1177/2042098617736422 29318002 PMC5753994

[B6] Bate A. Stegmann J. U. (2021). Safety of Medicines and Vaccines - Building Next Generation Capability. Trends Pharmacol. Sci. 42 (12), 1051–1063. 10.1016/j.tips.2021.09.007 34635346

[B7] Bodenreider O. (2004). The Unified Medical Language System (UMLS): Integrating Biomedical Terminology. Nucleic Acids Res. 32, D267–D270. Database issue. 10.1093/nar/gkh061 14681409 PMC308795

[B8] Bulcock A. Hassan L. Giles S. Sanders C. Nenadic G. Campbell S. (2021). Public Perspectives of Using Social Media Data to Improve Adverse Drug Reaction Reporting: A Mixed-Methods Study. Drug Saf. 44 (5), 553–564. 10.1007/s40264-021-01042-6 33582973 PMC8053157

[B9] Chen X. Faviez C. Schuck S. Lillo-Le-Louët A. Texier N. Dahamna B. (2018). Mining Patients' Narratives in Social Media for Pharmacovigilance: Adverse Effects and Misuse of Methylphenidate. Front. Pharmacol. 9, 541. 10.3389/fphar.2018.00541 29881351 PMC5978246

[B10] Code of Federal Regulations (2011). Title 21- Food and Drugs. Chapter I-Food and Drug Administration, Department of Health and Human Services. Subchapter D - Drugs for Human Use. Sec. 314.80 Postmarketing Reporting of Adverse Drug Experiences. [Online]. Available at: http://www.accessdata.fda.gov/scripts/cdrh/cfdocs/cfcfr/CFRSearch.cfm?fr=314.80 (Accessed Mar 16, 2022).

[B11] Correia R. B. Wood I. B. Bollen J. Rocha L. M. (2020). Mining Social Media Data for Biomedical Signals and Health-Related Behavior. Annu. Rev. Biomed. Data Sci. 3 (1), 433–458. 10.1146/annurev-biodatasci-030320-040844 32550337 PMC7299233

[B12] DuMouchel W. Pregibon D. (2001). Empirical Bayes Screening for Multi-Item Associations in Proceedings of the seventh ACM SIGKDD international conference on Knowledge discovery and data mining. San Francisco, California, Aug 26-29. 10.1145/502512.502526

[B13] European Medicines Agency (2017). Guideline on Good Pharmacovigilance Practices (GVP). Module VI – Collection, Management and Submission of Reports of Suspected Adverse Reactions to Medicinal Products (Rev 2). [Online]. Available at: http://www.ema.europa.eu/en/documents/regulatory-procedural-guideline/guideline-good-pharmacovigilance-practices-gvp-module-vi-collection-management-submission-reports_en.pdf (Accessed Mar 16, 2022).

[B14] Freifeld C. C. Brownstein J. S. Menone C. M. Bao W. Filice R. Kass-Hout T. (2014). Digital Drug Safety Surveillance: Monitoring Pharmaceutical Products in Twitter. Drug Saf. 37 (5), 343–350. 10.1007/s40264-014-0155-x 24777653 PMC4013443

[B15] Gartland A. Bate A. Painter J. L. Casperson T. A. Powell G. E. (2021). Developing Crowdsourced Training Data Sets for Pharmacovigilance Intelligent Automation. Drug Saf. 44 (3), 373–382. 10.1007/s40264-020-01028-w 33354751

[B16] Gendreau J. Ramsubeik S. Pitesky M. (2022). Web Crawling of Social Media and Related Web Platforms to Analyze Backyard Poultry Owners Responses to the 2018–2020 Newcastle Disease (ND) Outbreak in Southern California. Transbounding Emerg. Dis. [Epub ahead of print]. 10.1111/tbed.14454 35029049

[B17] Golder S. Smith K. O'Connor K. Gross R. Hennessy S. Gonzalez-Hernandez G. (2021). A Comparative View of Reported Adverse Effects of Statins in Social Media, Regulatory Data, Drug Information Databases and Systematic Reviews. Drug Saf. 44 (2), 167–179. 10.1007/s40264-020-00998-1 33001380 PMC7847442

[B18] Health Canada (2018). Reporting Adverse Reactions to Marketed Health Products. Guidance Document for Industry. [Online]. Available at: http://www.canada.ca/content/dam/hc-sc/documents/services/drugs-health-products/reports-publications/medeffect-canada/reporting-adverse-reactions-marketed-health-products-guidance-industry/reporting-adverse-reactions-marketed-health-products-guidance-industry.pdf (Accessed Mar 16, 2022).

[B19] Inácio P. Cavaco A. Airaksinen M. (2017). The Value of Patient Reporting to the Pharmacovigilance System: a Systematic Review. Br. J. Clin. Pharmacol. 83 (2), 227–246. 10.1111/bcp.13098 27558545 PMC5237689

[B20] Inch J. Watson M. C. Anakwe-Umeh S. (2012). Patient versus Healthcare Professional Spontaneous Adverse Drug Reaction Reporting: a Systematic Review. Drug Saf. 35 (10), 807–818. 10.1007/bf03261977 22928729

[B21] Jokinen J. D. Walley R. J. Colopy M. W. Hilzinger T. S. Verdru P. (2019). Pooling Different Safety Data Sources: Impact of Combining Solicited and Spontaneous Reports on Signal Detection in Pharmacovigilance. Drug Saf. 42 (10), 1191–1198. 10.1007/s40264-019-00843-0 31190237 PMC6739274

[B22] Kara V. Powell G. Merico E. Kaur N. Bate A. (2021). 20th ISoP Annual Meeting “Integrated Pharmacovigilance for Safer Patients” 8-10 November 2021 Muscat, Oman (Hybrid Meeting). Drug Saf. 44 (12), 1391–1470. 10.1007/s40264-021-01129-0 34751901 PMC8576318

[B36] Lee J. Y. Lee Y. S. Kim D. H. Lee H. S. Yang B. R. Kim M. G. (2021). The Use of Social Media in Detecting Drug Safety-Related New Black Box Warnings, Labeling Changes, or Withdrawals: Scoping Review. JMIR Public Health Surveill 7 (6), e30137. 10.2196/30137 34185021 PMC8277336

[B23] Li J. (2015). A Privacy Preservation Model for Health-Related Social Networking Sites. J. Med. Internet Res. 17 (7), e168. 10.2196/jmir.3973 26155953 PMC4526982

[B24] Li Y. Jimeno Yepes A. Xiao C. (2020). Combining Social Media and FDA Adverse Event Reporting System to Detect Adverse Drug Reactions. Drug Saf. 43 (9), 893–903. 10.1007/s40264-020-00943-2 32385840 PMC7434724

[B25] Makady A. Kalf R. R. J. Ryll B. Spurrier G. de Boer A. Hillege H. (2018). Social Media as a Tool for Assessing Patient Perspectives on Quality of Life in Metastatic Melanoma: a Feasibility Study. Health Qual. Life Outcomes 16 (1), 222. 10.1186/s12955-018-1047-z 30497502 PMC6267816

[B26] Nguyen T. Larsen M. E. O'Dea B. Phung D. Venkatesh S. Christensen H. (2017). Estimation of the Prevalence of Adverse Drug Reactions from Social Media. Int. J. Med. Inf. 102, 130–137. 10.1016/j.ijmedinf.2017.03.013 28495341

[B27] Platt R. Brown J. S. Robb M. McClellan M. Ball R. Nguyen M. D. (2018). The FDA Sentinel Initiative - an Evolving National Resource. N. Engl. J. Med. 379 (22), 2091–2093. 10.1056/NEJMp1809643 30485777

[B28] Rakhsha M. Keyvanpour M. R. Vahab Shojaedini S. (2021). “Detecting Adverse Drug Reactions from Social Media Based on Multichannel Convolutional Neural Networks Modified by Support Vector Machine”, in Proceeding of the 7th International Conference on Web Research ICWR, TEHRAN,Iran, 19-20 May. 10.1109/icwr51868.2021.9443128

[B29] Sarker A. Ginn R. Nikfarjam A. O'Connor K. Smith K. Jayaraman S. (2015). Utilizing Social Media Data for Pharmacovigilance: A Review. J. Biomed. Inf. 54, 202–212. 10.1016/j.jbi.2015.02.004 PMC4408239 25720841

[B30] Schachterle S. E. Tresnan D. Hauben M. Murugesan S. Sobel R. E. Bate A. (2019). Abstracts of the 35th International Conference on Pharmacoepidemiology & Therapeutic Risk Management, Pennsylvania Convention Center, Philadelphia, PA, USA, August 24‐28, 2019. Pharmacoepidemiol Drug Saf. 28, 980 Social media data analysis with population-based methods: A pilot investigation of mild hypersensitivity among users of subcutaneously administered monoclonal antibodies, 5-586. 10.1002/pds.4864 31429168

[B31] Stergiopoulos S. Fehrle M. Caubel P. Tan L. Jebson L. (2019). Adverse Drug Reaction Case Safety Practices in Large Biopharmaceutical Organizations from 2007 to 2017: An Industry Survey. Pharm. Med. 33 (6), 499–510. 10.1007/s40290-019-00307-x 31933240

[B32] Thomas M. Curry A. Painter J. L. Akhtar A. Schifano L. Powell G. E. (2017). “T-26: Case Study: Computing Complexity Scores to Identify Patients of Interest from Inspire.Com Forums for Safety and Beyond”, in Drug Information Association Annual Meeting, Chicago, IL, June 18-22.

[B33] Trigger S. Coleman B. (2019). Social Media Mentions of Electronic Nicotine Delivery Systems (ENDS) Battery-Related Overheating, Fires, and Explosions: Findings from a Pilot Study. Int. J. Environ. Res. Public Health 16 (8), 1308. 10.3390/ijerph16081308 PMC6517878 31013680

[B34] van Stekelenborg J. Ellenius J. Maskell S. Bergvall T. Caster O. Dasgupta N. (2019). Recommendations for the Use of Social Media in Pharmacovigilance: Lessons from IMI WEB-RADR. Drug Saf. 42 (12), 1393–1407. 10.1007/s40264-019-00858-7 31446567 PMC6858385

[B35] Wang R. Liu W. Gao S. (2016). Hashtags and Information Virality in Networked Social Movement. Online Inf. Rev. 40 (7), 850–866. 10.1108/OIR-12-2015-0378

