# Supplementary material for: Engaging Patients via Online Healthcare Fora: Three Pharmacovigilance Use Cases
Source: Front Pharmacol. 2022 Jun 3;13:901355. doi: 10.3389/fphar.2022.901355 (PMC9204179; doi:10.3389/fphar.2022.901355)
Supplement: Supplementary file 1 [file DataSheet3.docx]

***Supplementary Material***

**Figure S1.** Frequency of MedDRA high level group terms in posts from HealthUnlocked mentioning “denosumab” in the fora following mapping of adverse events to MedDRA preferred terms, and preferred term aggregation

MedDRA, Medical Dictionary for Regulatory Activities; NEC, not elsewhere classified.

**Figure S2.** Initial posts, follow-up information requested and provided to assess the ability to actively engage patients posting on social media fora (Inspire)

**
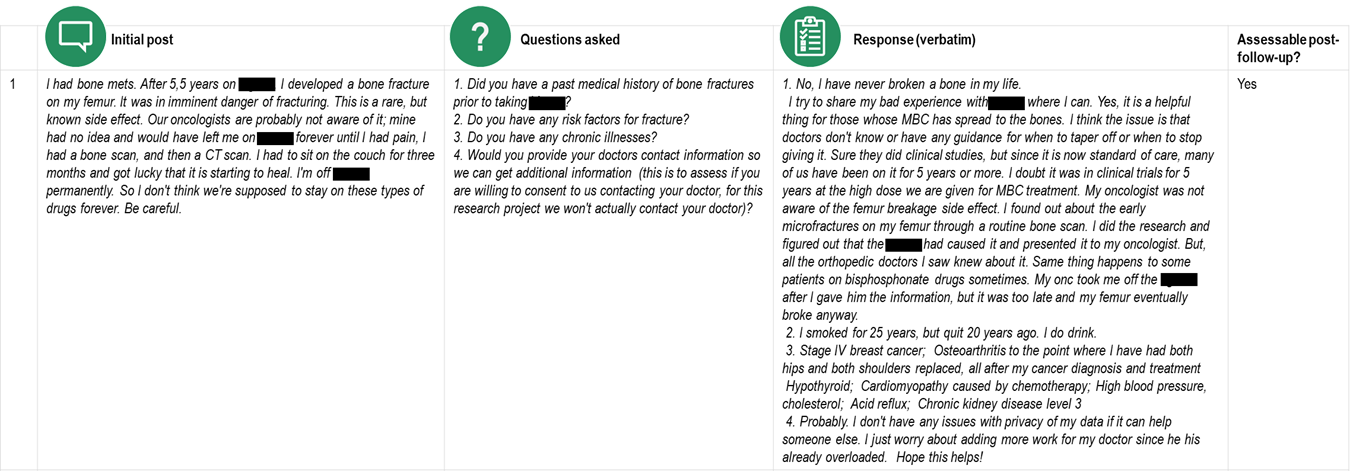
**


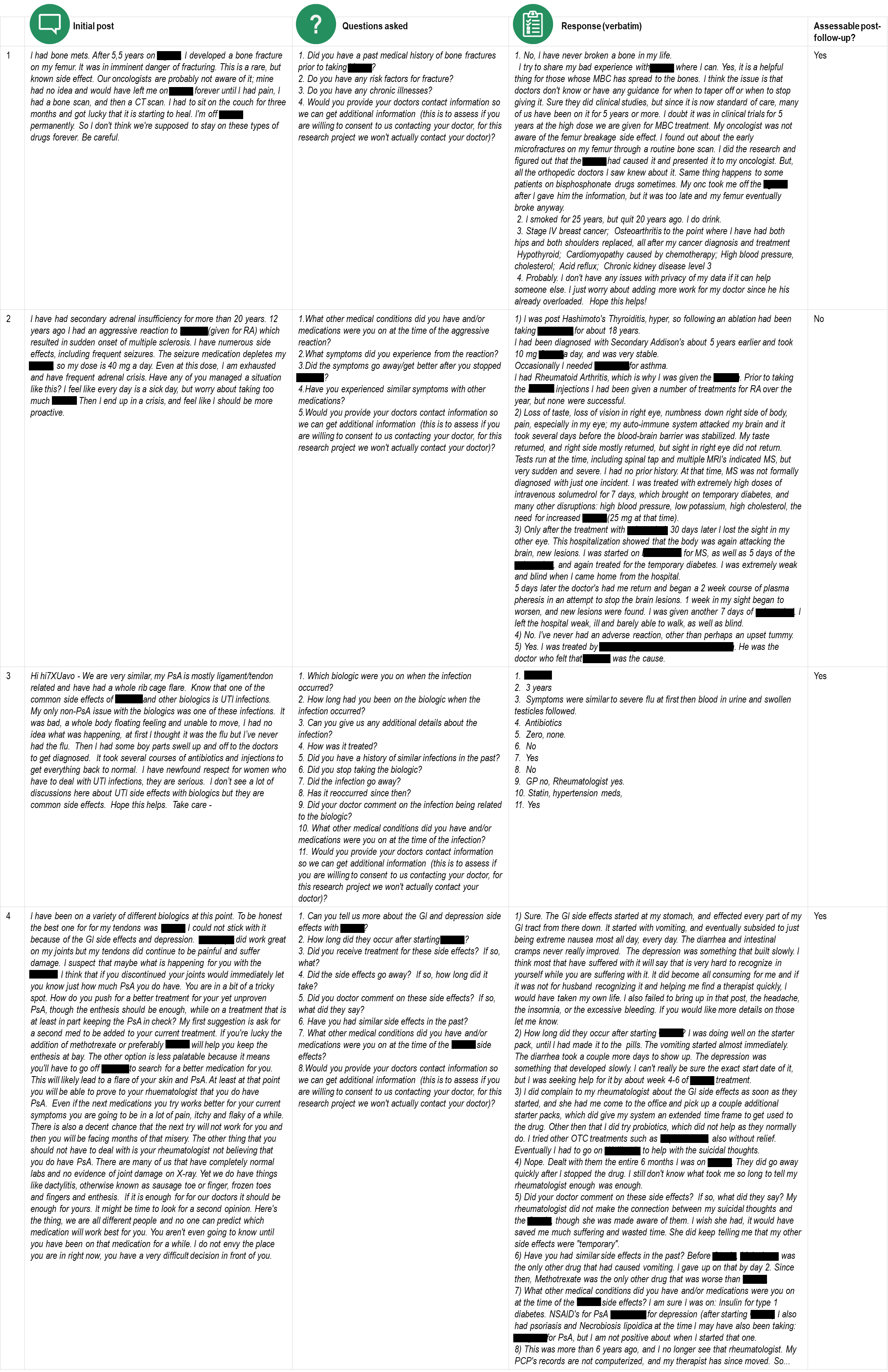

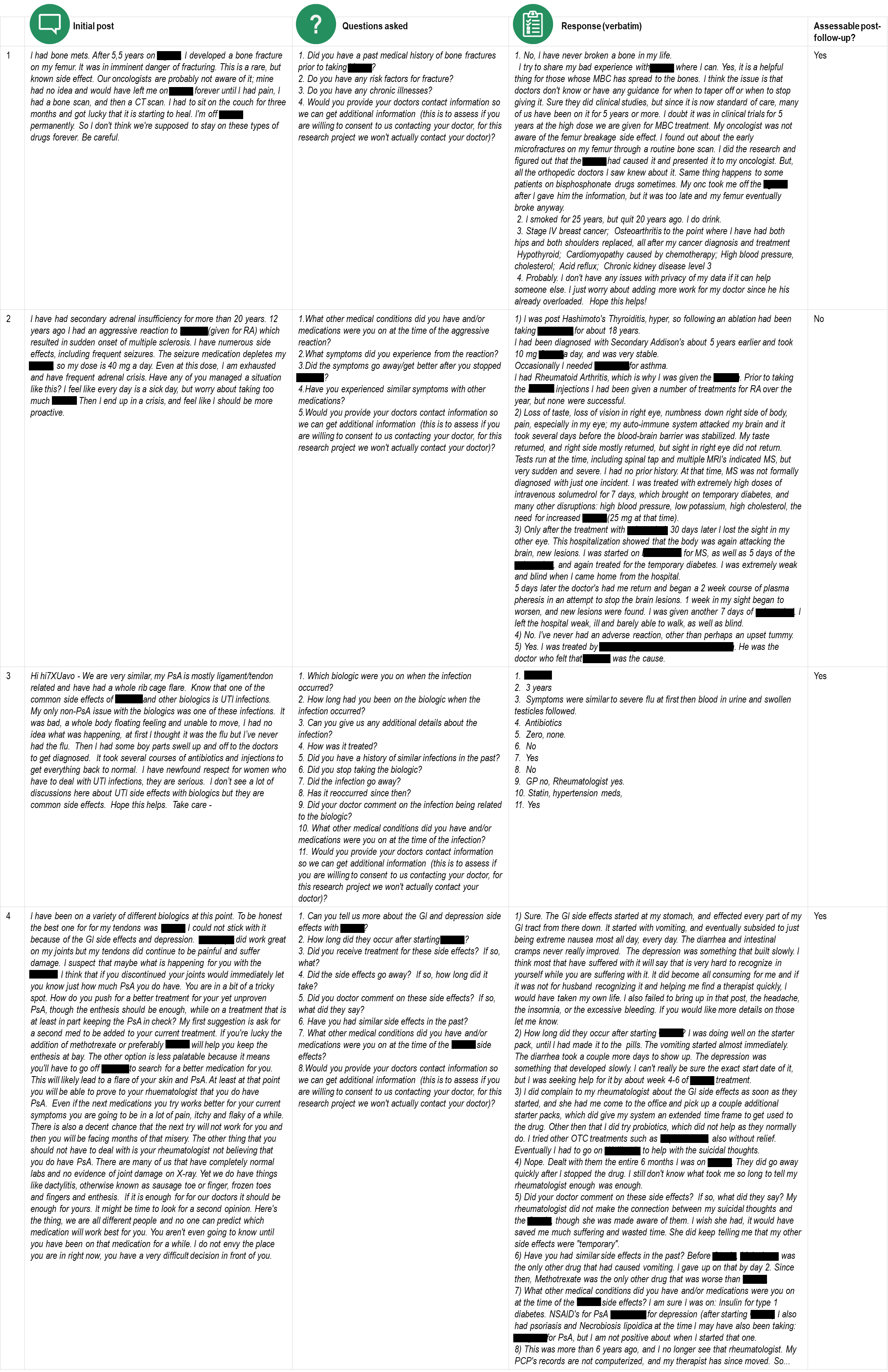

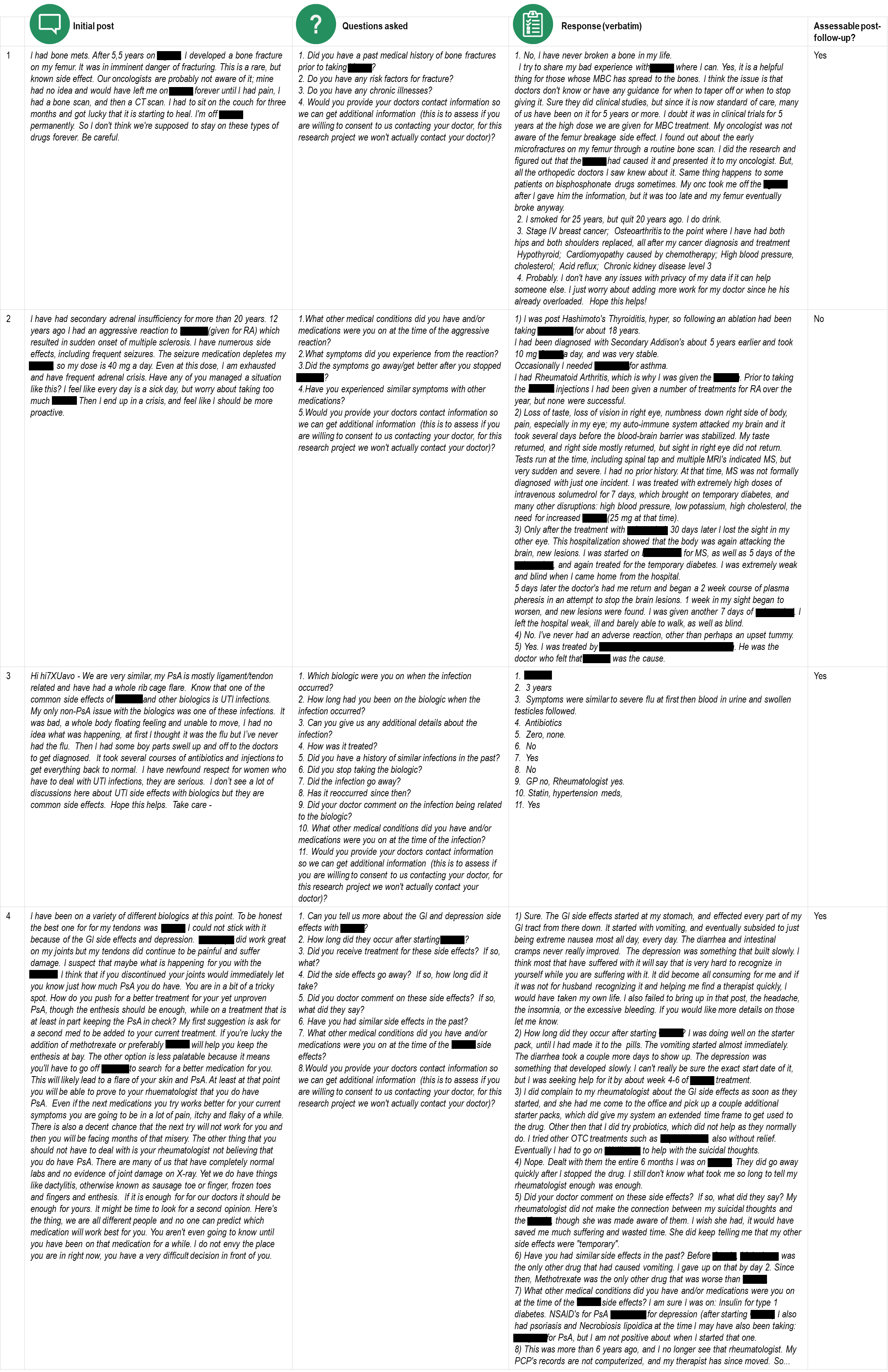


Note: Posts are listed verbatim but mentions to trade names of drugs or sensitive personal information have been redacted.

**Table S1.** Drug-event pairs used in the study

| **Drug** | **Date approved** | **Event of interest added in the product information** | **Information about event of interest in the product information at time of approval** | **Date event of interest added to in the product information** |
| --- | --- | --- | --- | --- |
| Denosumab, *Xgeva* | 11/18/2010 | Multiple vertebral fractures (MVF) following discontinuation (Sections 5.7, 6, 17) | Vertebral not in label; mentions pathological fractures | 1/24/2018 |
| Denosumab, *Prolia* | 6/1/2010 | Added multiple vertebral fractures (MVF) following discontinuation of Prolia (Sections 4, 5.6, 6.1, 6.2, 17.6) | Section 14.1 includes clinical study data on effect on vertebral fractures | 1/31/2017 |
| Pembrolizumab, *Keytruda* | 9/4/2014 | Added 5.6 Immune-Mediated Skin Adverse Reactions - includes SJS, TEN, exfoliative dermatitis, bullous pemphigoid | None in label, however exfoliative dermatitis was mentioned in Section 5.7 | 7/27/2017 |
| *Blinatumomab, Blincyto* | *12/3/2014* | *6.1 Added 'cranial nerve disorders (trigeminal neuralgia, trigeminal nerve disorder, sixth nerve paralysis, cranial nerve disorder, facial nerve disorder, and facial paresis)' under nervous system disorders* | *Not in label* | *11/30/2017* |
| *Daratumumab, Darzalex* | *11/16/2015* | *Updated 6.3 Postmarketing experience with newly identified risk of anaphylactic reaction and pancreatitis* | *Anaphylaxis and pancreatitis not mentioned* | *4/17/2020* |

SJS, Stevens-Johnson syndrome; TEN, toxic epidermal necrolysis.

Note: Rows in italic indicate drug-event pairs which could be used in the event that sufficient data was not available for denosumab and pembrolizumab.

**Appendix 1. Number and percentage of posts mentioning denosumab and adverse events by MedDRA PT (including adverse events of interest) stratified by quarter, by social media forum**

**Appendix 2. Number and percentage of posts mentioning pembrolizumab and adverse events by MedDRA PT (including adverse events of interest) stratified by quarter, by social media forum**
